# The value of supply chain coordination under moral hazard: A case study of the consumer product supply chain

**DOI:** 10.1371/journal.pone.0194043

**Published:** 2018-03-16

**Authors:** Yumi Lee, Sang Hwa Song, Taesu Cheong

**Affiliations:** 1 Graduate School of Logistics, Incheon National University, Incheon, South Korea; 2 School of Industrial Management Engineering, Korea University, Seoul, South Korea; Southwest University, CHINA

## Abstract

In this paper, we examine a real-world case related to the consumer product supply chain to analyze the value of supply chain coordination under the condition of moral hazard. Because of the characteristics of a buyback contract scheme employed in the supply chain, the supplier company’s sales department encourages retailers to order more inventory to meet their sales target, whereas retailers pay less attention to their inventory level and leftovers at the end of the season. This condition induces moral hazard problems in the operation of the supply chain, as suppliers suffer from huge returns of leftover inventory. This, in turn, is related to the obsolescence of returned inventory, even with penalty terms in the contract for the return of any leftovers. In this study, we show under the current buyback-based supply chain operation, the inventory levels of both the supplier and retailers exceed customer demand and develop vendor-managed inventory (VMI) system with base stock policy to remove any mismatch of supply and demand. A comparison of both systems shows that through the proper coordination of supply chain operations, both suppliers and retailers can gain additional benefits while providing proper services to end customers.

## Introduction

As companies have begun to focus more heavily on their core competencies and outsource non-critical processes to cost-efficient suppliers, supply chains have become more globally distributed and complex. Because it is difficult for both managers in distributed and independent ownership structures to manage the entire supply chain as an integrated system, companies tend to rely on contracts between participants within supply chains. When contracts properly provide incentives to participants, they can guide supply chain participants’ behaviors and align their goals and processes such that they are consistent with supply-chain-wide objectives. However, a supply chain consists of multiple independent organizations with diverse interests, each of which seeks to maximize its own profit, take advantage of the supply chain’s benefits, and minimize its own risks. So, in spite of the potential for contracts to incentivize participants to work together in a supply chain, incentive conflicts may hamper the ability of contracts to coordinate supply chains. Thus, no matter how well a contract is designed, it can have negative side effects.

There exists a notable example to illustrate unexpected implications of supply chain contracts. Blockbuster, a provider of home movie and video game rental services, successfully managed its supply chain through a revenue sharing contract with Hollywood movie studios [1, 2]. Before Blockbuster entered into the revenue sharing agreement with Hollywood studios, they purchased video tapes for about $65 and rented them to customers for about $3. However, movies at Blockbuster retail locations were often out of stock, as the company underestimated the initial demand for a film following its release on video (this demand sharply decreased within few weeks after the film’s release). Despite these stock-outs, Blockbuster remained reluctant to purchase video tapes to avoid them. The stock-outs were not desirable to movie studios, as the variable production cost for each copy of a film was negligible relative to the fixed production cost related to the production of the movie. So, the studios were incentivized to distribute as many videotapes as possible. Thus, rather than enter into a conventional wholesale contract (which was prevalent in the rental industry at the time), the movie studios proposed revenue sharing contracts to Blockbuster. After Blockbuster entered into a revenue sharing contract with the movie studios, the latter reduced the initial purchase price, which helped Blockbuster to stock its shops with more tapes, thereby reducing stock-outs. By sharing the risk associated with unsatisfactory demands, Blockbuster increased its market share from 25% to 31%.

Although the revenue sharing contract initially seemed to provide positive outcomes for both Blockbuster and the film studios, the video rental industry overlooked the issue of moral hazard and encountered a number of subsequent conflicts. These conflicts were epitomized the Walt Disney Company’s lawsuit against Blockbuster in the early 2000s. According to the July 3, 2003 Los Angeles Times, the Walt Disney Company accused Blockbuster of failing to account for missing video tapes, improperly charging for some promotional costs, and prematurely selling videotapes before the end of their useful rental lives. Although the revenue sharing contract between Disney and Blockbuster was terminated in 2001, Disney had no choice but to sue Blockbuster, since they failed to conclude agreement to avoid the misuse of the contract. The judge in the case ultimately ruled that Blockbuster must compensate Disney for the missing videotapes (of which there were hundreds of thousands in Blockbuster’s stock) and incorrect charges at the rate of $65 per tape.

Holmstrom [3] claimed that because individuals’ actions are often unobservable but nonetheless affect the outcome of business operations, moral hazard or incentive conflicts are caused by information asymmetry. He showed that problems associated with moral hazard or incentive conflicts intrinsic to contracts can be alleviated when an information system is available and facilitates the observation of agents’ actions with respect to the principal-agent relationship. In practice, however, it is difficult to enforce efficient system integration among supply chain partners, because companies are often reluctant to share their private information with other companies. Even in cases in which supply chain systems are integrated and partners share information with each other, it is often impossible to remove incentive conflict completely. In trying to increase short-term profits, companies may attempt to disguise information or share incomplete or distorted data with other companies. Therefore, when a supply chain is fragmented and operates as a function of multiple independent companies’ participation, it is inevitable that moral hazard and incentive conflicts will emerge.

In this paper, we present a case study to illustrate the unexpected behaviors that can result from a supply chain contract. Specifically, we explore the implications of a buyback contract that is fully favorable to buyers. In a buyback contract structure, both suppliers (sellers) and retailers (buyers) can achieve positive outcomes by sharing the risks associated with mismatching supply and demand. The re-purchase of a portion of unsold inventories by suppliers can effectively reduce the risk of a mismatch between supply and demand, thereby motivating a retailer to order more inventory than it otherwise would have. This type of agreement reflects a fundamental philosophy of sharing both profits and risks as a means to achieve the benefits of the supply chain. However, the case study presented here illustrates that when a supplier focuses on its own revenue in a buyback contract, it may sabotage coordination across the supply chain and result in substantial leftover inventory. To illustrate this point, we compare the practices inherent to buyback contracts with a vendor-managed inventory system to show the waste that results from a lack of coordination.

The rest of the paper is organized as follows: (1) we first review studies that have explored moral hazard in the context of supply chain contracts; (2) we then describe a real-world case study in which moral hazard affects the supply chain. Specifically, moral hazard from both the retailer’s and supplier’s perspective will be explained in detail; (3) we introduce the vendor managed inventory (VMI) system with a base-stock policy as a means to coordinate the supply chain. This section will also feature an analysis of how much the supply chain has lost and the degree to which it can be coordinated, and (4) we finally discuss the implication of our findings.

## Literature review

One of the most important issues related to supply chain management is the coordination or maintenance of balance among the supply chain’s participants. This coordination or balance is largely a function of determining how to allocate the chain’s total profits to each member. In this paper, we evaluate incentive conflicts and information asymmetry as factors that affect the balance of supply chains. Early research on supply chain coordination largely focused on the mismatch between supply and demand resulting from information distortion and the bullwhip effect [4]. Since then, subsequent research has explored methods for resolving problems associated with a lack of coordination and information sharing [5–8].

Some researchers have suggested that supply chains can be efficiently coordinated, even when subject to incentive conflicts or information asymmetry. Simatupang and Sridharan [9] explored how to coordinate decentralized supply chains that are subject to incentive conflicts to maximize profits among the chain’s constituents. They argued that managerial inertia-which comes from inappropriate performance measures, outdated policies, information asymmetry, and misalignment of incentives-disturbs supply chain cooperation. To reduce managerial inertia (and therefore improve cooperation), they proposed a number of interrelated and cooperative processes that individual companies in the supply chain can implement simultaneously to produce a cohesive and collaborative supply chain. In another line of research, Corbett [10] investigated efficient inventory management in the presence of incentive conflicts and under conditions of information asymmetry. More specifically, he measured costs using a principal-agent model to evaluate coordination inefficiency. Moreover, he introduced a consignment stock to test a case with a four-week long production cycle and two weeks of safety stock and compared the supplier’s set up cost and the buyer’s backorder cost due to stock-out. The order lot size and reorder point was determined by the (*Q*, *r*) model. Ultimately, Corbett [10] suggested that consignment stock helps a supplier to reduce the batch size while the retailer increases safety stock.

Bolandifar et al. [11] sought to identify a buyer’s optimal contract strategy under conditions of information asymmetry related to the supplier’s cost structure and actions taken to avoid capacity risk. To do so, they used a case supply chain with a long production lead-time. When production lead-time is long, there exists an incentive conflict whereby the supplier seeks to maintain capacity to avoid extra investment, and the buyer expects the supplier’s capacity to be more flexible to satisfy uncertain demand. The study by Zhou et al. [12] specifically sought to explore supply chain coordination in the context of consumer learning. When the supply chain introduces an innovative product to the market, it is subject to consumer uncertainty, as the value of the product is unknown to consumers shortly after its release. They explored coordination among suppliers, retailers, and consumers, investigated firms’ marketing efforts, and evaluated the effects of consumer learning. They also explored the role of information and double moral hazard given consumer learning. They argued that a firm’s marketing efforts decrease as consumers become aware of a product’s value. One interesting note that emerged from this study is that information asymmetry provides benefits to both suppliers and retailers, given consumer learning. This finding is unique in the literature on supply chain management. Furthermore, Gao et al. [13] considered single and double moral hazard models with quality level uncertainties and proposed a partial cost allocation contract which can fairly allocate defective costs.

A contract is an efficient method for coordinating a supply chain. Cachon [14] examined various supply chain contract types in an effort to determine which is most effective in coordinating a supply chain and which is most flexible, as well as whether it is worth adopting a newsvendor model. Wholesale price contracts are commonly used in practice, but fail to increase supply chain inventory. In contrast, buyback contracts allow for the return of a retailer’s unsold goods to the supplier at the end of a selling season. This encourages the buyer to order more stock at a reduced risk. So, buyback contracts are superior to wholesale price contracts as a means to coordinate and achieve benefits for supply chain participants. Cachon [15] also explored the coordination of supply chains when suppliers and retailers have different levels of inventory risk. Specifically, he evaluated push contracts, pull contracts, and a hybrid of these two-called advance purchase discount contracts. In exploring these different types of contracts, Cachon [15] found that advance purchase discount contracts help to coordinate a supply chain most efficiently, as the inventory risk associated with it is shared between retailers and suppliers. Huang et al. [16] studied a contract mechanism to mitigate moral hazards in a virtual enterprise setting and showed that a suitable monitoring strategy can reduce the moral hazard effectively. Yoo et al. [17] investigated pricing and return policies in a closed-loop supply chain with a powerful supplier. They compared three contract mechanisms including wholesale, buy-back and quantity discount contracts and provided decision guidelines to minimize the retailer’s moral hazard.

Taken together, the literature suggests that a supply chain system should be integrated to allow for better information sharing, and contracts should be designed to align the incentives of the supply chain members’ incentives. Although we explore issues similar to those addressed in past literature, we focus more closely on the role of moral hazard, which has yet to be studied comprehensively. Through an illustrative case study, we illustrate the value of supply chain coordination when issues related to moral hazard arise.

## Case study: Moral hazard in a supply chain contract

In this case study, the supplier is a local branch of a renowned multinational corporation. This supplier imports most products and materials from worldwide production facilities and distributes finished and semi-finished consumer products to domestic retailers. Though the parent company has a good reputation around the world, the supplier is currently struggling to catch up to the market leader in South Korea. Because the number of stock keeping units (SKUs) in the Korean market is large (i.e., there are a number of competing brands and product specifications), the supply chain is volatile in terms of demand uncertainty. Relative to the general commodity market, the market of focus is comparatively small. However, competition is quite fierce; substitute products of similar quality are available from multiple competitors. Owing to low brand loyalty and high competition within the industry, it is essential for companies to increase the availability of their products to consumers so they can access them without delay. Attempting to increase their products’ availability can complicate management of the supply chain for some companies. It is quite difficult for a retailer to maintain large stocks, as the market is characterized by significant demand uncertainty and a large number of product SKUs.

To ease the burden on retailers due to the maintenance of large inventories, some suppliers enter into buyback contract schemes in which retailers can return unsold inventory to the supplier at any time, or exchange that inventory for other products. To avoid violations of the contract, suppliers constrain the conditions under which retailers can return or exchange their stock. For example, when retailers return or exchange their inventory, they must purchase 30% worth of new products relative to the products they returned. At the outset, the initial contract structure looks promising, as it allows the retailer to order a greater number of products from the supplier (at a lower risk), thereby maintaining high levels of inventory and making products immediately available to consumers. Suppliers can also benefit from the contract, as initial sales revenue will increase as a function of a greater number of orders from retailers. Moreover, the additional 30% that retailers are required to purchase when they exchange or return items can also increase sales revenue. Increased product availability can decrease the revenues lost due to missed sales. In spite of the benefits associated with this contract structure, this supply chain contract type can cause later problems related to moral hazard. Because retailers can return stock whenever necessary, they tend to worry less about forecasting demand and managing inventories. In addition, suppliers’ sales departments encourage retailers to engage in problematic buying behavior, which exacerbates the aforementioned problems. Because sales are the key performance indicator for sales departments, unnecessarily inflated orders are beneficial even if the buyer incurs increased risk of return of leftover stock (which can generate an additional 30% worth of revenue for them). In this way, suppliers can easily increase sales to retailers, even if sales to final customers do not increase.

### Supplier’s inventory status

**Fig 1:**
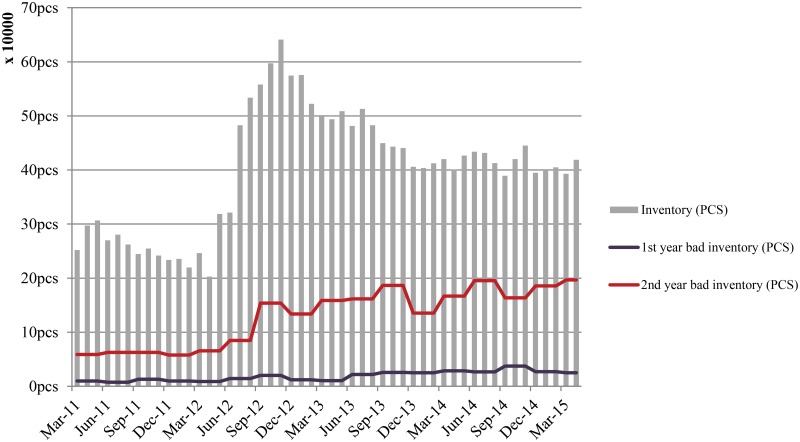
Supplier’s inventory status.

Suppose a retailer carries an old product X, and that a new product Y is about to be introduced into the market. Under the buyback contract structure, the supplier’s inventory of X will increase due to continuous returns and exchanges, but Y will be distributed to the market upon its release. Fig 1 illustrates the supplier’s inventory over five years. The grey bars signify the supplier’s on-hand inventory at the end of each month, and the lines in the figure indicate obsolete inventories. Until the middle of 2012, the supplier’s end-of-month inventory was less than 300,000 units, but that level increased drastically each month between July 2012 and November 2012. Because the supplier introduced Y in March of 2012 and subsequently approved requests for returns and exchanges of X, the supplier’s on-hand inventory increased considerably until the end of 2012. In July of 2012, another new product Z was introduced aiming to occupy low price market. These new product launches induced the retailers to sell back leftover stock, thereby increasing the supplier’s inventory. To illustrate, consider that the ratio of obsolete inventory to all inventory was less than 25% in March of 2011, but after the introduction of Y and Z, this ratio had grown to higher than 50%. Although the supplier facilitated the availability of products for retailers to provide to end users, the retailers tended to order more than necessary, even when penalized with a mandatory purchase of 30% more goods from the supplier. Retailers believed that they could return obsolete leftover stock to the supplier, and therefore did not manage their obsolete stocks. The supplier will later be forced to salvage the outdated products at a lower price, negatively affecting the company’s bottom line. In this way, a focus on short-term sales revenue will eventually hurt the supplier’s profitability.

**Fig 2:**
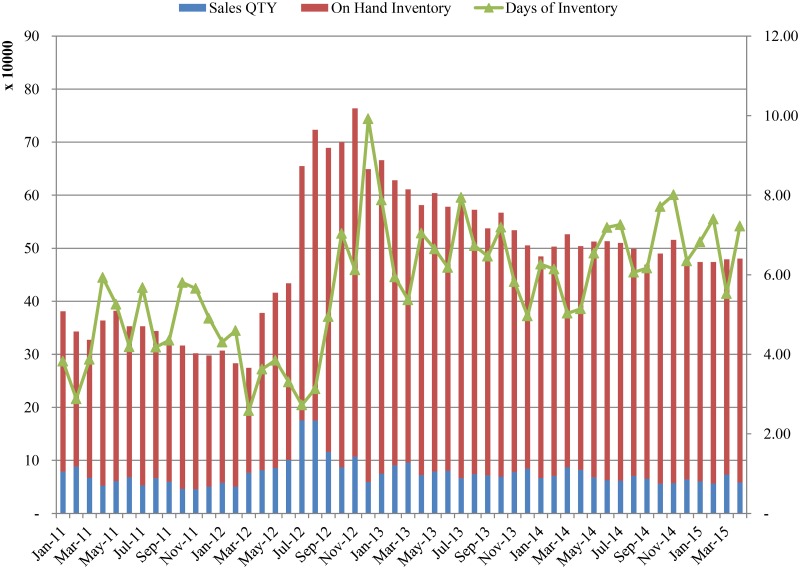
Months of inventory at the supplier.

Fig 2 illustrates the supplier’s inventory status in terms of inventory coverage. When the supplier launched Y and Z in 2012, its inventory coverage dropped below four months. However, sales volume decreases caused inventory coverage to increase sharply at the end of that year. This indicates that the supply chain failed to synchronize sales and inventory management properly.

**Fig 3:**
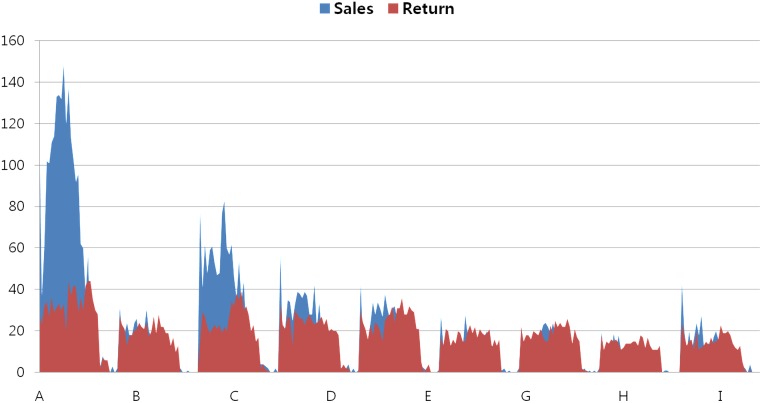
Demand distribution key by SKU.

In looking at the details associated with stock returns, it is clear that sales and returns differ across SKUs. The side-effect of the buyback contract was more pronounced for products for which there was low demand. As shown in Fig 3, sales are larger than returned leftovers for high-demand products. However, most low-demand products were returned to the supplier by the retailer. The buyback contract induced greater sales of low-demand products to retailers (sell-in orders), but demand for the product was not sufficiently high among end users to increase sales to those customers (sell-out orders). Therefore, in supply chains characterized by a large number of SKUs, buyback contracts can bring supply and demand into imbalance.

**Fig 4:**
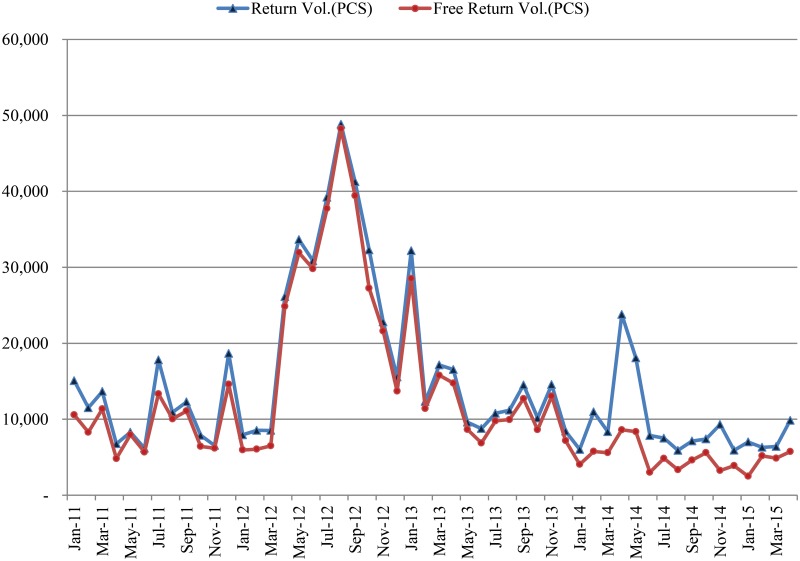
Retailer returns and exchanges.

As shown in Fig 4, many retailers demonstrate similar return and exchange patterns. Since the introduction of Y into the market, the number of returns that the retailer requested increased between April 2012 and December 2012. In this period, retailers returned many of their leftovers and exchanged them with new products, suggesting that retailers attempt to maintain a steady inventory portfolio. When new products are introduced to the market, retailers automatically exchange their old products for them. Without a well-structured supply chain management system, problems related to a mismatch between supply and demand will only get worse. For instance, most unsold goods will be unable to be resold to the market. As a consequence, nearly 40% of returned products become excessive inventory that the company is unable to sell, even two years after its return.

Given the current description of how the supply chain is managed, the supplier is unable to access, monitor, or control retailers’ inventories, despite the fact that suppliers must nonetheless approve returns and exchanges. This is largely due to retailers selling suppliers’ products, which they can return if the products go unsold. Given this reduced risk, retailers often fail to attend to overstock inventory. The inventories of large retailers that operate under a franchise company are often controlled by the franchise itself. However, there exist hundreds of small retailers that employ a very small number of people. These small retailers tend not to engage in inventory management. Moreover, retailers rarely exhibit loyalty to any brands, as alternative products from other suppliers may provide them with more favorable financial outcomes. Given these market conditions, suppliers must provide retailers with quality products and customer service. As outlined above, limited market growth hampers suppliers’ ability to obtain their rivals’ market share. Still, suppliers seek to achieve increased sales and revenue growth each year. For example, the supplier seeks to increase sales by 7% each year through aggressive marketing and sales activity, in spite of worsening market conditions. The moral hazard may comes from the aggressive sales activity to fill a revenue gap arising from the optimistic sales target. In this paper, to address issues related to moral hazard, we explore the vendor managed inventory system with base stock level as a means to reduce a supplier’s excess inventory.

## Value of supply chain coordination

### Base-stock based coordination

**Fig 5:**
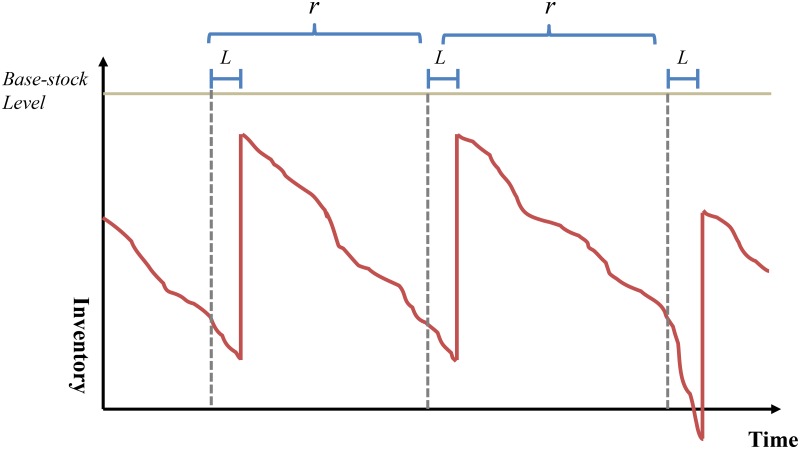
Illustration of base-stock policy.

To evaluate the issues outlined above, we tested a vendor-managed inventory system (VMI) with base stock levels (specifically, base-stock policy) to minimize the potential mismatch between supply and demand, thereby keeping inventory levels low and availing products to the consumers that demand them from retailers. We remark that base-stock policy [18], which is one of well-known periodic review replenishment policies in inventory theory, specifies the decision rule if there exists a quantity called *base stock level* such that the stock level after ordering for every review period is as close to the base stock level as possible [18] and is illustrated in Fig 5.

**Fig 6:**
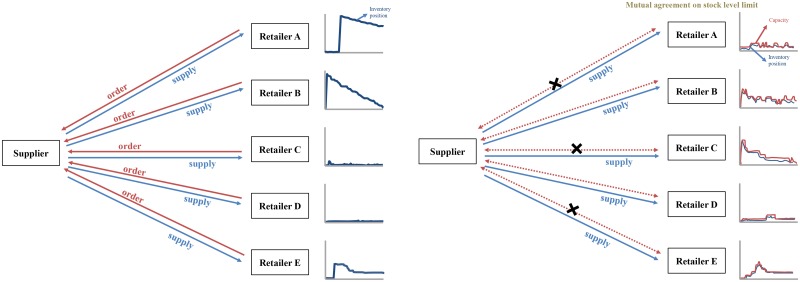
Current system (left) vs. VMI with base stock policy (right).

In the VMI system, inventory is controlled by suppliers, not retailers. Specifically, suppliers can restrict the maximum capacity of their inventories, base-stock level, to be held by each retailer. To manage backorders, the suppliers pay penalties when retailers encounter back orders and sends them to the retailer in an expedited transportation mode. The terms of the mutual agreement between a supplier and retailer allows for the regular adjustment of the latter’s base stock level. The nature of the VMI system is depicted visually in Fig 6.

The current buyback contract structure mandates that retailers order goods from a supplier that is not privy to information concerning consumer demand for those goods. The only information available to suppliers is the retailers’ order histories. Under the VMI system, information concerning consumer demand is shared among supply chain participants. So, suppliers can forecast sales based on consumer demand, and supply only the items necessary to retailers to satisfy this demand. If demand is greater than the retailer’s base stock level, the retailer back orders the goods.

There exists some research that serves as the basis for our analysis in supply chain coordination. For example, Gavirneni et al. [19] investigated the supply chain relationship between a supplier and a retailer connected via the (*s*, *S*) policy (also known as the base stock policy). More specifically, they explored the benefits of information flow in the context of the (*s*, *S*) policy. In their experiments, under the (*s*, *S*) policy, inventory holding costs fell while inventory availability increased. Cachon [20] evaluated the VMI system as a means to coordinate a supply chain comprised of a supplier and multiple retailers. He demonstrated that the VMI system allows suppliers to effectively adjust reorder points as a function of their recognition of increasing demand. Yao et al. [21] argued that the VMI system reduces costs for both suppliers and retailers. Because the reorder point is directly linked to consumer demand, we adopt the base stock policy to analyze the supply chain’s performance. The parameters are given in Table 1.

**Table 1:** Parameters.

Notation	Explanation
*r*	length of the review period
*L*	lead time
*AVG*	average daily demand
*STD*	standard deviation of daily demand
*z*	standard score or *z*-score (from standard Normal distribution) [22]
$z\times STD\times \sqrt{r+L}$	safety stock

### Case study

**Fig 7:**
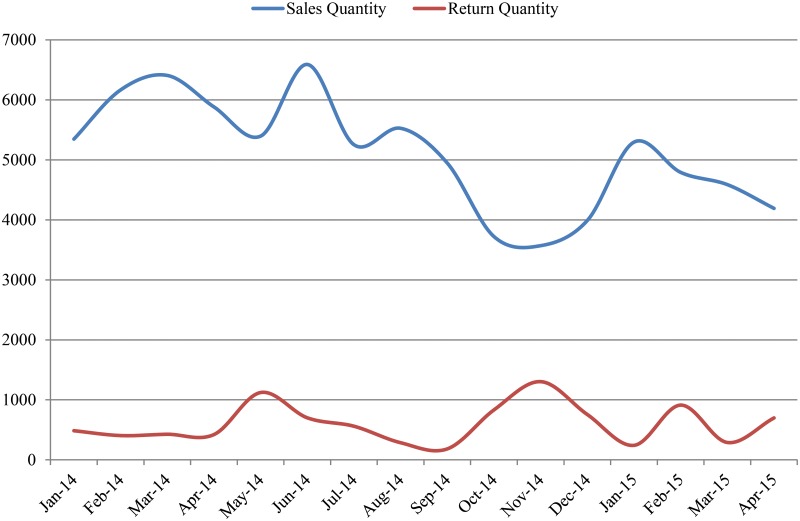
Sales and returns of product N.

In this section, we apply the aforementioned framework of a vendor-managed inventory system (VMI) with base stock policy to the moral hazard issues in the supply chain of interest. To evaluate the base stock policy for the supplier’s DC (Distribution Center), we selected one of the supplier’s products, N. N is one of the Y products the supplier introduced to the domestic market in 2012. Demand for N has decreased from 2014 to the middle of 2015 (see Fig 7). Although sales for N has gradually declined, returns have been relatively stable. Given this, we assume that N will soon be obsolete, and the supplier will control the retailer’s inventory to predict future returns and exchanges.

First, we examine how the base stock policy has improved the supplier’s inventory performance. Parameters for doing so were chosen on the basis of current business practices.

- *r* = 3 weeks
- *L* = 3.5 weeks
- *AVG* = average 3 weeks demand
- Service level = 99.9%

**Fig 8:**
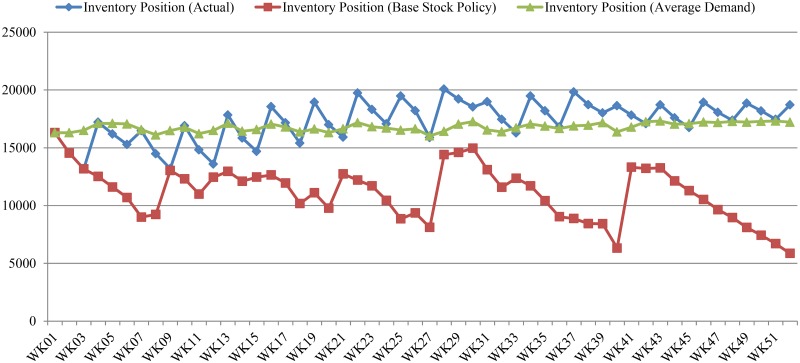
Comparison of inventory position.

In practice, the supplier seeks to replenish its stock through orders to a manufacturer every three weeks (based on on-hand inventory, sales history, and sales forecasts). Results indicate a substantial difference between the supplier’s current inventory management performance and the performance of the base stock policy (see Fig 8).

The line with diamonds represents the supplier’s inventory position under current replenishment system with buyback contract. The line with squares indicates the supplier’s prospective inventory position under the VMI system with base stock policy. Finally, the line with triangles indicates the supplier’s prospective inventory position under the VMI system, but with replenishment orders contingent on average demand. As illustrated in Fig 8, it seems that the base stock policy can assist suppliers to reduce their inventories. More specifically, under the base stock policy, the supplier’s DC can reduce its inventory position such that it is closer to the average monthly demand for N. More detailed quantitative results associated with each of the systems are outlined in Table 2.

**Table 2:** Inventory performance with actual product cost.

	Current System	Average Demand Policy	Base Stock Policy
Inventory Position	17,270 pcs	16,798 pcs	11,097 pcs
On Hand Inventory	13,724 pcs	13,227 pcs	8,206 pcs
Inventory Holding Cost	KRW 57.5M	KRW 55.4M	KRW 34.4M
Inventory Turnover	3.6 times	3.9 times	5.6 times
Replenishment Order Quantity	63,651 pcs	63,085 pcs	50,801 pcs

On the basis of these evaluations, we assert that the VMI system with the base stock policy offers significant benefits to the supplier in terms of inventory performance. First, the policy allows the supplier to optimize its inventory and inventory turnovers. Second, it helps the supplier to reduce unnecessary purchases so as to reduce holding costs (by roughly 40%) relative to the current system. Third, the base stock policy can also reduce unnecessary inventory at the parent company’s global production facilities.

In spite of these benefits, there may be some reasons why retailers prefer to carry their initial inventory. Retailers leverage price discounts (ranging from 10% to 30%) on large replenishment orders if they purchase the initial inventory of new products, and some retailers are afraid to lose sales even if the supplier promises to deliver the requested products within 48 hours. Moreover, when retailers can return leftover stock when they purchase new goods, they tend to ignore their inventory. To evaluate the overall effect associated with the adoption of the VMI system, we also examine the retailer’s inventory performance. In doing so, it is possible to identify how the base stock policy prevents the wasting of cash on the part of the retailers. For this analysis, we propose that the retailers are managed by the supplier under the VMI system using the base stock level as the reorder point. To simulate the implementation of the VMI system across different retailers, we selected four retailers (respectively called A, B, C, and D) that have different consumer demands and purchasing behaviors. The details associated with these retailers are presented in Table 3.

**Table 3:** Retailer order history.

Type of Inventory	Retailer A	Retailer B	Retailer C	Retailer D
Initial Purchased Quantity of New Product	121pcs	314pcs	94pcs	0pcs
Total Purchased Quantity at the End	585pcs	585pcs	346pcs	251pcs
Real Customer Demand	464pcs	271pcs	252pcs	251pcs

The data showed that with the exception of the smaller retailer, D, retailers purchase more than necessary and return their leftovers. In contrast, Retailer D did not return any leftovers and not place an initial order for the new product. Instead, to reduce inventory and maximize cash flow, Retailer D ordered items only when necessary. Using these data, we implemented another base stock policy with the following conditions.

- *r*: a day
- *L*: a day
- Service level: 99.9%

Results for the one-year data are summarized in Table 4. Table 4 indicates that Retailers A and B may carry too excess inventory. This may be due to the fact that (a) they overestimate consumer demand, or (b) do not pay attention to their inventory because they can easily return leftover stock to the supplier. Instead, Retailers A and B may make large initial inventory purchases to secure price discounts from the supplier. Finally, the results demonstrate that Retailers A, B, and C purchase too much initial inventory. Retailer B, in particular, can reduce his carrying inventory by almost 85% if it adopts the VMI system. Though Retailer D did not return any leftover stock, its operations hurt the supplier, since product availability is low. As such, the Retailer D may be unable to fulfill all consumer demands; consumers may then seek out substitute items from competitors. In either case, the supplier will lose customers.

**Table 4:** Inventory results: Base stock policy for retailers.

Retailer	Initial Order under the Current System	Max Inventory Position by Base Stock Policy
A	121pcs	72pcs
B	314pcs	49pcs
C	94pcs	82pcs
D	0pcs	49pcs

We also explored the benefits of the retailer’s adoption of the base stock policy and Table 5 summarizes the results.

**Table 5:** Retailer inventory performance.

Retailer	Inventory Holding Cost under the Current System	Inventory Holding Cost under the Base Stock Policy	Obsolescence Cost at the Supplier under the Current System	Obsolescence Cost at the Supplier under the Base Stock Policy	Return Amount to Supplier under the Current System^★^
A	1.05	0.62	0.16	0.09	1.36
B	2.71	0.42	0.41	0.06	3.52
C	0.81	0.71	0.12	0.11	1.06
D	-	0.42	-	0.06	-
Total	4.57	2.18	0.69	0.33	5.94

(Unit: KRW Million, Selling Price: KRW 8,644/pc)

^★^ Under the VMI system, leftovers at the end of the period are salvaged by the supplier. Therefore, return is zero under the VMI system.

As indicated by the data in Table 5, retailers can save between KRW 0.01 million and 2.3 million in inventory holding costs. Because the VMI system with a base stock policy maintains low inventories, retailers pay less for the products in their stock. In addition, if retailers return unsold goods to the supplier, suppliers shall compensate for the returned goods for between KRW 1.06 million and 3.52 million. Moreover, under the VMI system with a base stock policy, the supplier’s obsolescence cost is much lower than the obsolescence cost incurred in the current system. The base stock policy prevents foolish cash management practices on the part of the retailers. As a result, we conclude that the base stock policy can help members of a supply chain to reduce resource waste and improve cash flows. Under the new VMI system, the retailer’s profits are higher than in the current system. These results are outlined Table 6 as well.

**Table 6:** Retailer profits.

Retailer	Demand (pieces)	Revenue^♣^ (KRW)	Cost under Current System (KRW)	Cost under VMI System (KRW)	Improvement (%)
A	464	25.9M	5.18M	4.40M	15%
B	271	15.0M	5.55M	2.55M	54%
C	252	13.9M	3.20M	2.64M	18%
D	251	14.0M	2.24M	2.38M	-0.06%

^♣^ Revenue is the same under both systems since we do not allow for lost sales. Orders exceeding carried inventory are back-ordered.

With a retailer’s selling price of KRW 55,000/pc and a product cost of KRW 8,644/pc, our results suggest that retailers can generate greater profit under the VMI system rather than carrying initial inventory. Retailer B, who carried excessive initial inventory, was able to increase profit by more than 20% relative to the current system.

## Concluding remarks

In this paper, we analyze the value of supply chain coordination under the condition of moral hazard. Due to the unique characteristics of the buyback contract scheme, retailers fail to pay attention to their inventory levels of leftovers at the end of the season. However, the sales departments of suppliers often encourage retailers to order more product to increase their sales. This tension induced infused the supply chain with issues related to moral hazard. As a result, the supplier was forced to accept substantial returns of leftover inventory, much of which was fast-approaching obsolescence. We showed that under the current buyback-based supply chain operations, retailers maintain inventories that exceed real customer demand. In response to this shortcoming of the current system, we developed the VMI system with a base stock policy and compared it to the current system using real data gathered from current business practices. The results of our analyses suggested that by coordinating supply chain operations, both suppliers and retailers can enjoy greater profits and provide quality services to their customers.

Although the supply chain contract currently employed in the case study contains penalties for the return and exchange of leftover inventory, retailers nonetheless fail to pay attention to their inventory and therefore, fail to match supply to demand for their goods. This causes a great deal of frustration for suppliers, who lose sales because of this inattention. The supplier’s sales department would like to increase product availability in the market to increase the overall sales, thereby improving performance on a KPI. However, the supplier’s operations department was responsible only for returned leftovers and obsolete inventory. This moral hazard issue can hurt the supplier’s profitability in the long run.

The VMI system with base stock policy helps to coordinate the supply chain and increase profits for both suppliers and retailers. Moreover, the new VMI system is better for customers, as retailers do not need to manage or handle their holding inventory or the timing of their reorders. We predict that through the VMI system, suppliers can increase revenues and market share in the long-term. The VMI system even accounts for retailers’ fears of losing customers due to a lack of control over orders and management of their inventory; suppliers guarantee a 99.9% service level for all retailers under the VMI system. Moreover, expedited delivery service is guaranteed for back orders. One disadvantage associated with the adoption of this system, however, is that the supplier will suffer a short-term revenue loss due to fewer orders from retailers. However, in the long-run, suppliers’ profits will increase.

Finally, we propose that the sales department’s sales targets should be adjusted in accordance with sales to the final customer. The current KPI for the sales department is based exclusively on sales to the retailer rather than the customer. This motivates suppliers to inflate sales by pushing products onto retailers, and guaranteeing 100% returns of any leftovers. One method for resolving this issue is to reflect leftover returns from retailers in the sales KPI. More specifically, whenever a sales department’s KPI is evaluated, loss from leftover returns should be deducted from the retailers’ sales revenue.

## Supporting information

S1 File De-identified data set used for analysis. (XLSX)
